# Doege-Potter Syndrome and Pierre-Marie-Bamberger Syndrome in a Patient With Pleural Solitary Fibrous Tumor: A Rare Case With Literature Review

**DOI:** 10.7759/cureus.7919

**Published:** 2020-05-01

**Authors:** Anup Solsi, Karen Pho, Shiva Shojaie, Dawood Findakly, Tarreq Noori

**Affiliations:** 1 Internal Medicine, Creighton University Arizona Health Education Alliance/Valleywise Health Medical Center, Phoenix, USA; 2 Internal Medicine, University of Arizona College of Medicine, Phoenix, USA

**Keywords:** solitary fibrous tumor, doege-potter syndrome, pierre-marie-bamberger syndrome

## Abstract

Solitary fibrous tumors (SFT) represent a unique subset of mostly benign heterogeneous tumors with mesenchymal cell origins. These tumors have been reported in the past as being mostly indolent, with a slowly evolving clinical course and low potential for malignancy. Although found systemically, the incidence of SFT arising intrathoracically, from the pleura of the lung, is relatively poorly documented in the medical literature. SFT is a rare phenomenon, but in even rarer circumstances, these tumors are associated with distinctive paraneoplastic syndromes, such as Pierre-Marie-Bamberger syndrome (PMBS) and Doege-Potter syndrome (DPS). PMBS presents as digital clubbing and hypertrophic pulmonary osteoarthropathy. DPS has been characterized as a non-islet cell tumor hypoglycemia due to the ectopic secretion of insulin-like growth factor 2 (IGF-2), a pattern seen in fewer than 5% of cases of SFT. Treatment is typically through surgical resection. In our research of the medical literature, we found only very few cases in which the association with SFT and both paraneoplastic syndromes were described. Here, we report an uncommon case of a 68-year-old male patient found to have an incidental right hemithoracic tumor with digital clubbing and intermittent severe episodes of fasting hypoglycemia after initially presenting with a syncopal episode.

## Introduction

The first known case of solitary fibrous tumor (SFT) was described in 1870 by Wagner and was further characterized by Klemperer and Rabin in 1931 as either a benign, localized mesothelioma or a metastatic, diffuse mesothelioma [1,2]. It was not until the 1980s, when advancement in technology allowed immunohistochemical methods to identify the cell origins of SFT as being derived from the mesenchymal cells that were deposited subpleurally, rather than from the pleural mesothelium lining itself [2,3]. Primary pleural tumors, usually diagnosed at comparable rates in men and women in their 6th or 7th decade of life, consist of fewer than 5% of all pleural tumors and less than 2% of all soft tissue tumors [4]. Most of these tumors present asymptomatically, with pulmonary lesions found incidentally through various imaging modalities. Those who present with symptoms often have dyspnea or atypical chest pain most commonly, but in rare instances, hemoptysis and obstructive pneumonitis from airway obstruction has been identified [1]. In uncommon scenarios, SFT presents with a paraneoplastic syndrome, the most well documented being Pierre-Marie-Bamberger syndrome (PMBS), which is the most common, and Doege-Potter syndrome (DPS). PMBS manifests as digital clubbing, long bone periostitis, and arthritis due to hypertrophic osteoarthropathy from bone surface and soft tissue calcification [5]. The presentation of this syndrome is consistent with non-specific arthritic type symptoms such as joint stiffness, swelling, arthralgias and pain along the long bones.

DPS occurs when a large SFT ectopically excretes Insulin-like growth factor 2 (IGF-2), resulting in a type of hyperinsulinemic hypoglycemia [6]. DPS was first exclusively described in 1930 by Doege and Potter as a non-islet cell tumor hypoglycemia [7]. The incidence of DPS is so rare that there have never been any systematic studies published with regards to detailing this subtype of solitary fibrous tumors [8].

The following report attempts to discuss the current understanding of SFT, its relationship with DPS and PMBS and recommendations for future investigation through the presentation of a unique case.

## Case presentation

**Figure 1:**
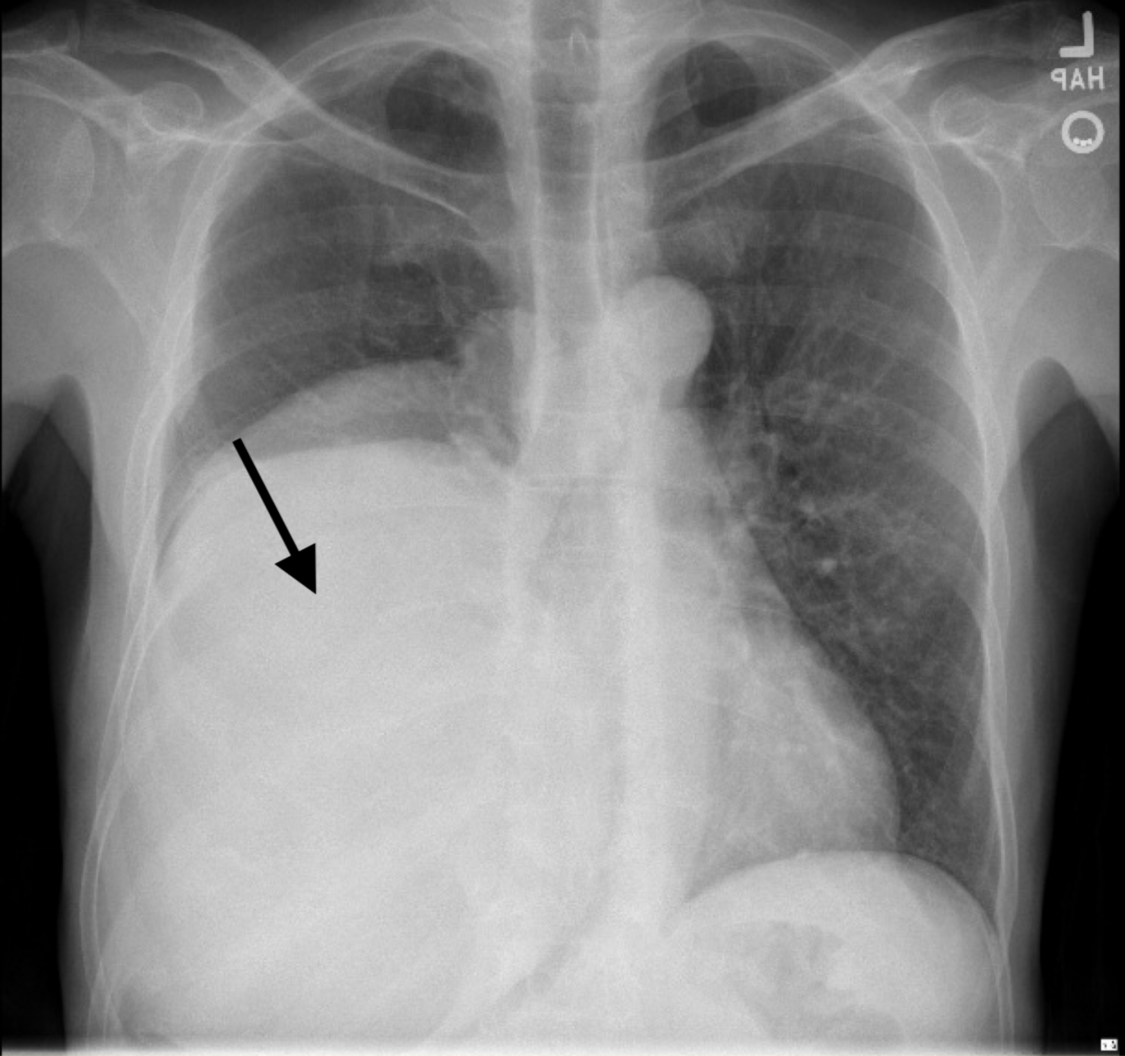
Chest X-ray showing right lower lung diffuse opacity/mass (arrow). The opacity displaces the heart posteriorly, extends inferiorly below the field of view. The opacity obscures the right heart border and right hemidiaphragm.

A 68-year-old Hispanic male with no significant medical history presented to the emergency department after an unwitnessed syncopal episode while walking to the restroom. The clinical review revealed a chronic non-productive cough, but no symptoms of dyspnea, chest pain, or weight loss. He endorsed a brief history of smoking for approximately 4-5 years (one pack every two days) but quit over 20 years ago. Physical exam revealed a cachectic appearing gentleman, with digital clubbing in bilateral digits and toes. On examination of the lungs, there was severely diminished breath sounds in all right lung fields, dullness to percussion, and no superficial lymph node enlargement. Cardiovascular and abdominal examinations were unremarkable. Routine laboratory studies were drawn, a complete blood count revealed no anemia, and complete metabolic panel returned with electrolytes within the normal ranges. A chest X-ray was obtained, showing a right lower lung diffuse opacity of unclear origin, likely a mediastinal mass vs. pulmonary mass (Figure 1).

**Figure 2:**
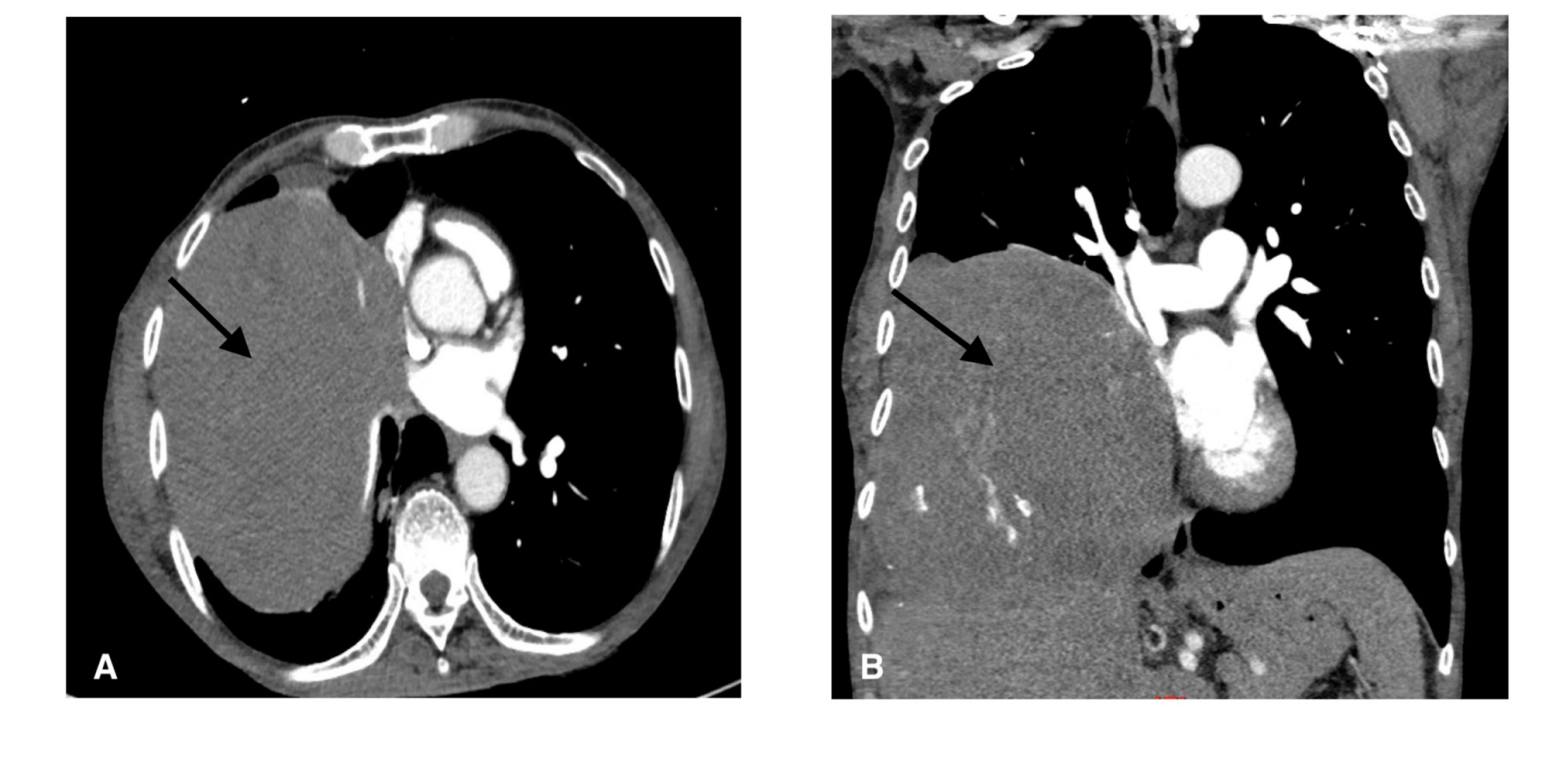
(A) Cross-sectional image of CT chest demonstrating 14 x 16 x 18 cm heterogenous right lung/thoracic mass (arrow) arising from right hilum/middle mediastinum or right hemidiaphragm. (B) Coronal CT scan showing right lung mass (arrow).

For further characterization, a CT chest with contrast was ordered, demonstrating a 14 x 16 x 18 cm heterogenous right lung/thoracic mass arising from right hilum/middle mediastinum or right hemidiaphragm with mediastinal lymphadenopathy (Figure 2A, 2B). When questioned about the lung mass, the patient revealed he had known this 20 years earlier when it was the size of a quarter and biopsy performed approximately 15 years ago in Mexico was reportedly benign.

**Figure 3:**
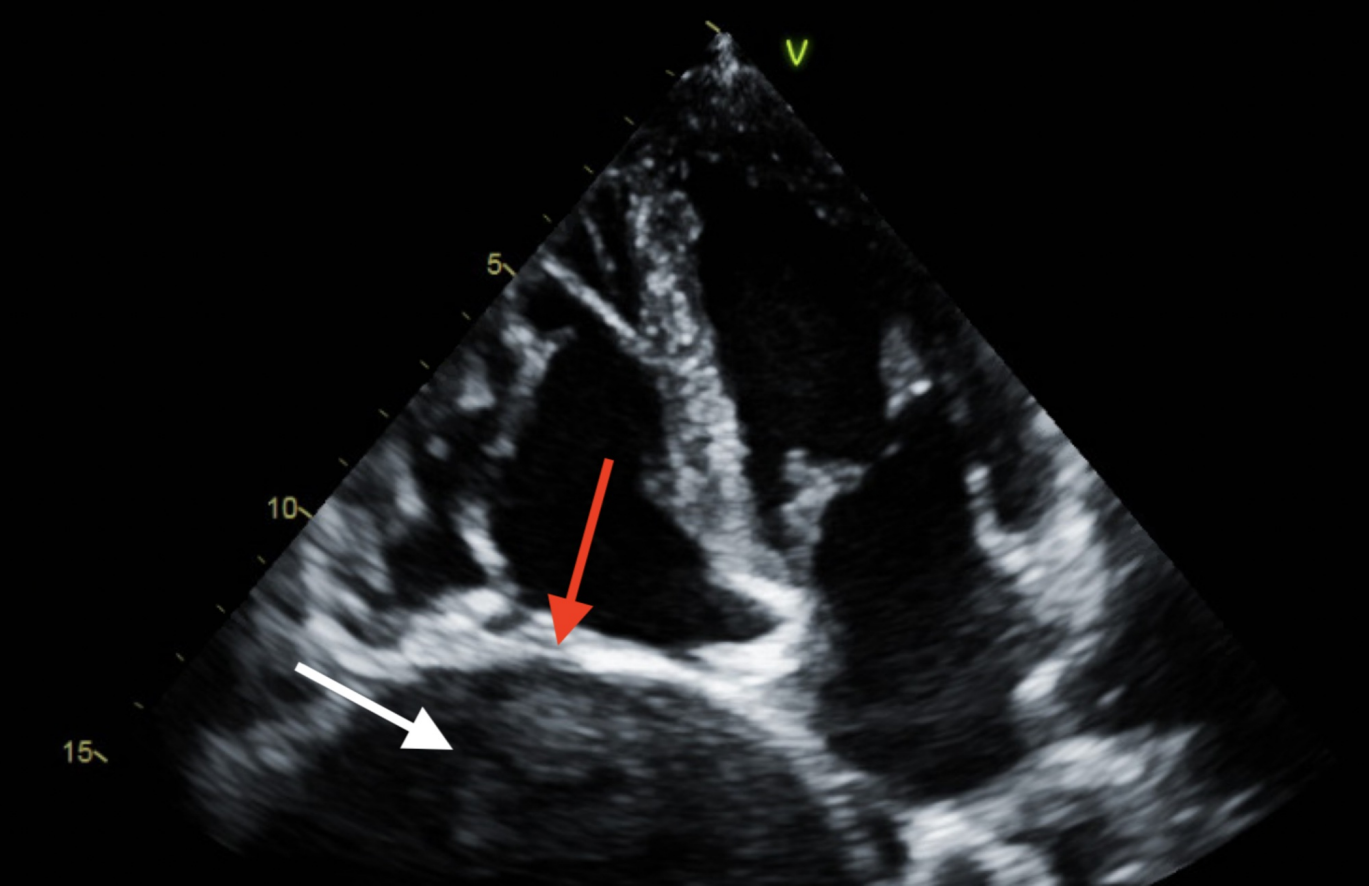
Transthoracic echocardiogram showing lung mass (white arrow) compressing the right atrial cavity (red arrow).

To determine the origin of this tumor, a CT-guided needle biopsy of the lung was performed. Tissue samples were sent for miscellaneous cultures with gram stain, AFB stain, and AFB culture, as well as fungal culture; all were negative, with no organisms or polymorphonuclear leukocytes found. Tissue specimen was sent to pathology for interpretation. Transthoracic echocardiography was ordered to rule out a cardiogenic etiology of his syncopal episode, showing a large mass compressing the right atrial cavity (Figure 3).

While the patient was hospitalized, morning glucose on the third day showed a critical value of 42 mg/dL, though the patient had eaten his previous meal. Interestingly, all hypoglycemic episodes thereafter were fasting. Further workup ruled out secondary causes of hypoglycemia. A morning cortisol 11.66 mg/dL, C-peptide 4.0 ng/mL, fasting insulin 21 uIU/mL, and Insulin-like growth factor 1 (IGF-1) of 73 ng/mL, were measured. The patient initially required IV Dextrose to maintain blood sugars within the acceptable range.

**Figure 4:**
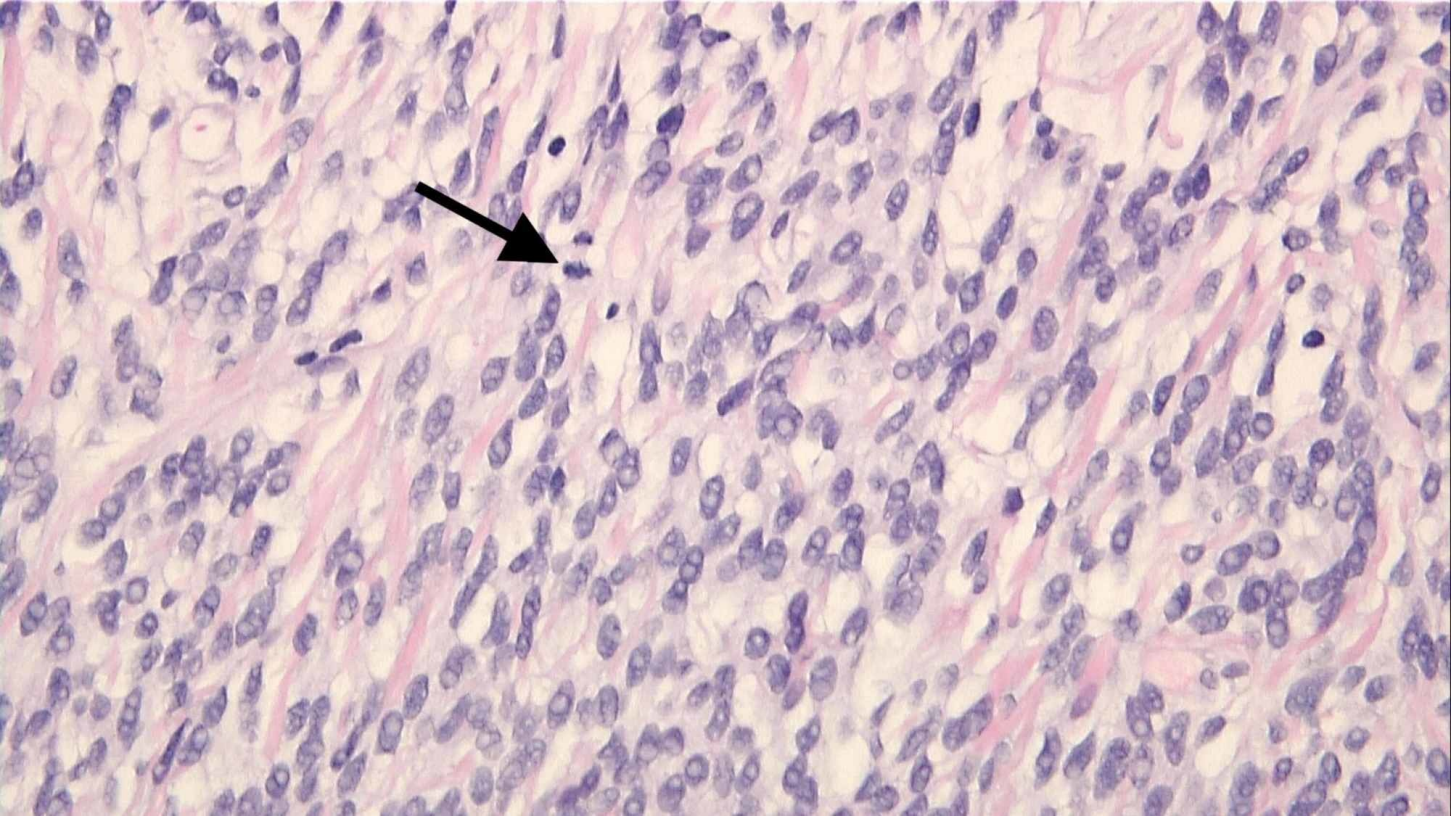
Histochemical analysis showed a spindle cell lesion composed of sheets of spindle cells with oval or elongated nuclei separated by collagen. Focal areas with mitotic figures up to 3 per 10 HPF were identified (arrow).

**Figure 5:**
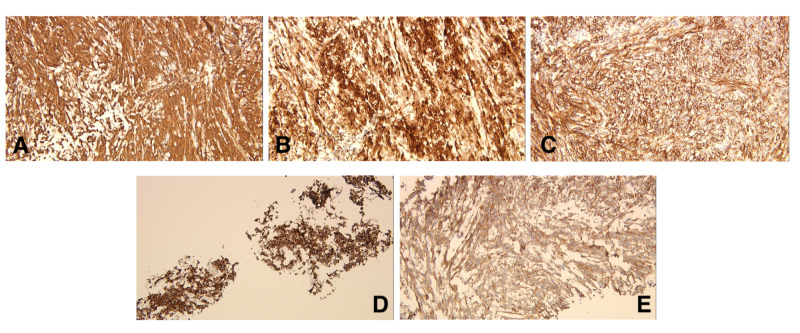
Immunohistochemistry showing positive stains for (A) Vimentin (B) Desmin (C) CD34 (D) BCL-2 (E) CD99.

**Figure 6:**
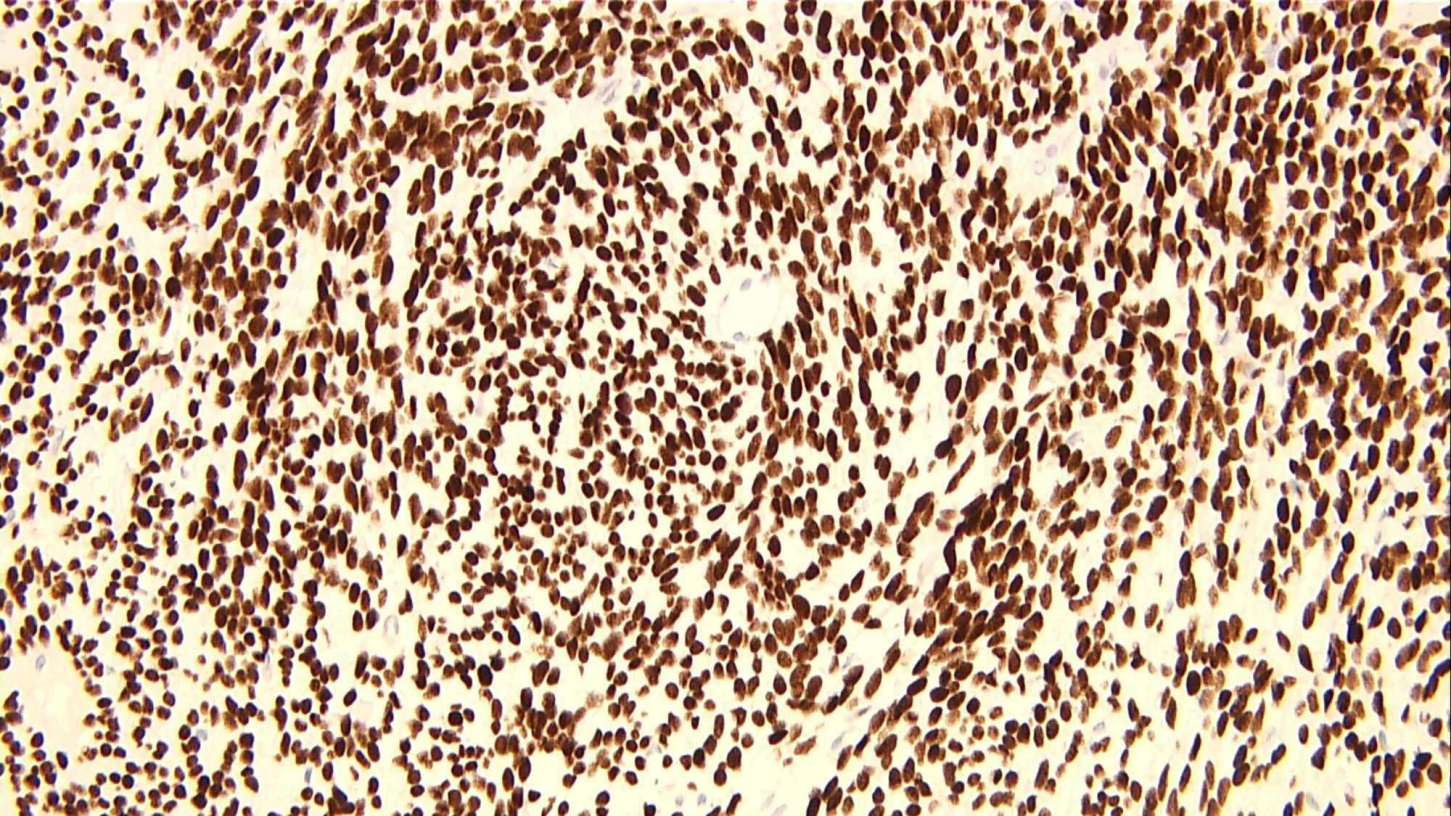
STAT6 showed diffuse strong positive nuclear staining.

Final tissue diagnosis of SFT with high risk for aggressive behavior was made. Histochemical analysis showed a spindle cell lesion composed of sheets of spindle cells with oval or elongated nuclei separated by collagen. Focal areas with mitotic figures up to 3 per 10 HPF were identified as were foci of necrosis and hyalinization (Figure 4). The spindle cells stained positive with vimentin (Figure 5A), Desmin (Figure 5B), CD34 (Figure 5C), BCL2 (Figure 5D), and CD99 (Figure 5E). Stains for EMA, S-100, CD5, CD117, SMA, and melanin were negative. Immunostains for beta-catenin, TLE1, and STAT6 were additionally performed. Beta-catenin was negative, and TLE1 shows patchy rare positive cells. STAT6 showed diffuse strong positive nuclear staining in the tumor (Figure 6). The immunohistochemical features were consistent with a diagnosis of a solitary fibrous tumor.

At this stage, the clinical diagnosis was confidently identified as being DPS. Endocrinology was consulted regarding further investigation and management. Based on prior studies, the team recommended initiating Prednisone 30 mg daily to prevent hypoglycemic episodes prior to definite treatment of surgical resection. Previous literature reviews have indicated this tumor has a well-known clinical correlation of hypoglycemia secondary to IGF-2 release. IGF-2 was ordered, returned at 63 ng/mL. After consideration of the final pathology and the entire clinical picture, vascular surgery determined due to the highly aggressive nature of the tumor, surgical resection was not appropriate at this time and thought the patient would benefit from radiation to shrink the tumor prior to removal. Oncology was asked to assist with the case and recommended outpatient chemotherapy with Mesna, Doxorubicin, Ifosfamide, Dacarbazine (MAID) to possibly debulk the tumor to a size where it may stop secretion of IGF-2. The patient did not receive any chemotherapy while hospitalized.

A few weeks after discharge, the patient presented to the oncology office to discuss further treatment options. Since discharge, he had done well with no symptoms. Chemotherapy was again discussed with the patient and its potential to decrease tumor size to make it amenable for resection. Instead of the MAID regimen discussed in the hospital, the patient agreed to proceed with a regimen of Gemcitabine and Paclitaxel (GemTaxol), which he is currently receiving.

## Discussion

SFT with associated hypoglycemia is a uniquely uncommon occurrence, with some estimates suggesting only 48 cases published since 1981 [9]. These tumors vary in the site of origin, from the pleural cavity, mediastinum, pericardium, retroperitoneal spaces, liver, thyroid, orbit, bladder, intestines, and soft tissues [1,8]. Although the tumor can present in a broad range of ages, the peak age is between the sixth and eighth decades of life, during which hypoglycemia manifests itself the most [9]. Symptoms can be thought of as intrathoracic vs. extrathoracic. Intrathoracic symptoms such as dyspnea, cough and hemoptysis result from tumor growth suppressing lung tissue as well as from pleural effusions [10]. On the other hand, extrathoracic symptoms are due to the paraneoplastic syndromes. Hypoglycemia has many presentations, such as dizziness, lightheadedness, diaphoresis, anxiety, palpitations and syncope. It can often be the initial indicator that suggests a diagnosis of SFT [8]. Additionally, symptoms of clubbing, finger pain/paresthesias, joint pain and bony changes as a component of PMBS due to hypertrophic osteoarthropathy, can be seen as the first clinical sign of SFT [11]. These extrathoracic manifestations have been reported to resolve within hours to days after total resection of the tumor and have been reported to recur if the tumor recurs, which can make these clinical signs an imperative marker for remission status.

Amongst the patients with pleural SFT, PMBS has reported a prevalence of 35%, whereas DPS is seen from 2-4% of patients, most commonly in the right pleural cavity with sizes in excess of 20 cm [5,9,12]. Imaging studies are routinely performed early in the evaluation of intrathoracic or intraabdominal disease processes when SFT is first detected. A chest X-ray may show an opacity or consolidation of uncertain origin within the thoracic cavity. More specifically, pleural SFT may appear as a well-circumscribed, lobular, solitary nodule or mass in the lung periphery, usually in close approximation to the pleural surface [13]. Subsequently, CT scans are obtained to reveal the full extent of the tumor; still, however, it is difficult to differentiate between benign and malignant SFT. CT typically shows a low-density lesion (25-40 Hounsfield units), but the presence of atelectasis, central necrosis, or an inhomogeneous tumor image can muddy the process of discerning SFT [14]. The characteristics consistently seen on CT that are later identified as malignant SFTs include lobulated borders, large tumor size, calcifications, and ipsilateral pleural effusions. Other diagnostic imaging modalities include MRI, 18-fluorodeoxyglucose-positron emission tomography and bronchoscopy.

The mechanism of hypoglycemia in SFT has previously been described. The release of an unprocessed or incomplete high molecular weight form of IGF-2 has the ability to activate insulin receptors, causing termination of hepatic gluconeogenesis and increased uptake of peripheral glucose, both of which contribute to hypoglycemia [15]. This form of IGF-2 can further bind to IGF-1 receptors and suppress the release of insulin and IGF-1 [16]. It is worth noting that not all SFT have elevated serum concentrations of IGF-2, as was shown in a prior report where just 80% of SFTs expressed IGF-2 [17].

What exact physiological mechanism is responsible for PMBS is not well understood, however abnormal synthesis of hepatocyte growth factors and/or excessive hyaluronic acid from the tumor are postulated theories [18]. Moreover, paraneoplastic secretion of VGEF and PDGF cytokines may also play a role.

Histochemical analysis for SFT will ordinarily depict multiple patterns of spindle-shaped cells with varying amounts of cellularity. SFTs often stain positive for CD34, BCL2, CD99, and vimentin, but this is not necessarily a highly specific association [19]. A positive STAT6 is usually most indicative of SFTs (>95% correlation), which was seen in our patient [2]. Furthermore, negative stains for AE1/AE3, synaptophysin, S-100, and calretinin were shown to be reliable indicators for SFT [8].

Currently, the gold-standard therapy for SFT and symptoms from DPS and PMBS is radical resection of the tumor, with or without adjuvant therapy. The type of surgical resection performed depends on multiple factors such as the location of the tumor, size of the tumor, and degree of invasion into surrounding structures; nevertheless, an aggressive surgical approach is recommended for both benign and malignant tumors due to high likelihood of recurrence [1]. Prior studies have demonstrated the complete resolution of hypoglycemia and finger clubbing in almost all instances following surgical resection, although these may recur if lesions reappear [5]. Recurrence rates have been documented to be around 2-4% for benign SFTs, but up to 68% for malignant tumors with specific histological characteristics [9].

The role of adjuvant and neoadjuvant chemotherapy and radiation therapy has not been well studied in patients with SFTs. Currently, the common practice is to employ chemotherapy and radiation therapy when a patient is deemed to be an unsuitable surgical candidate, if there is incomplete resection or a histologically malignant tumor. It can be safely assumed that patients with large tumors with invasive properties or those with multiple co-morbidities are not ideal surgical candidates, and alternative management should be pursued. Our literature search did not reveal a previously studied chemotherapy or radiotherapy regimen for patients with SFT, likely contributing to mixed recommendations regarding their use. The notion that SFTs could be chemotherapy-resistant is a possibility, but further investigation into this is required.

Another critical aspect of management of SFT is the treatment of hypoglycemia in patients with DPS. Initially, when the diagnosis was unclear, our team chose to manage hypoglycemia with IV Dextrose (50% dextrose in water) 25 mL injections as needed, then D5W-NS (5% dextrose in normal saline) infusions to adequately control the blood sugars. For the management of hypoglycemia in SFT, no previous studies have shown sufficient glucose management using dextrose infusions. Instead, glucocorticoids have been shown to be the most effective treatment of hypoglycemia through suppressing the production of IGF-2, as seen in previous studies where IGF-2 levels reduced as much as 75% in response to steroids [20]. We agree appropriate glucocorticoid regimens include prednisolone 25 mg/day, dexamethasone 2.0 mg/day, or methylprednisolone 32 mg/day. For our patient, prednisone 30 mg/day was started once the diagnosis was confirmed, but the patient continued to have low glucose levels in the 70s mg/dL, requiring an increase to 40 mg/day, giving an excellent response. Steroid dosing can be difficult in this circumstance, our recommendation is to have a patient dependent approach.

As our patient was deemed to be a poor surgical candidate due to the size and aggressive nature of his SFT, he was initiated on chemotherapy. Currently, he is undergoing combination chemotherapy with gemcitabine and paclitaxel, a regimen traditionally used for breast cancer, bladder cancer, and non-small cell lung cancers. If a favorable response is observed with a decrease in tumor size and subsequently a reduction in IGF-2 and cytokine secretion, perhaps further chemotherapy and radiotherapy regimens can be studied to combat these tumors to give non-surgical candidates a better prognosis.

## Conclusions

Solitary fibrous tumors have been described in medical literature as mostly indolent tumors with low malignant potential. In very rare instances, as in this case of a 68-year-old male, these tumors are associated with two distinct paraneoplastic syndromes, Pierre-Marie-Bamberger syndrome and Doege-Potter syndrome. Although diagnosis is made through IGF-2 levels and tissue biopsy with special stains, it is crucial to remember a normal IGF-2 does not rule out the diagnosis of DPS. Radical resection of the tumor has been shown to resolve the symptoms associated with these paraneoplastic syndromes, however, medical management prior to surgery is still a debated topic. We recommend management of hypoglycemia using glucocorticoids, choosing a regimen based on patient factors and response. Further research is necessary to explore other alternatives to glucocorticoid therapy in treating hypoglycemia-associated DPS, especially in patients with a contraindication to glucocorticoids. Furthermore, deeper investigation is needed to identify the potential role of combination chemotherapy regimens as an alternative to surgery in poor surgical candidates.

## References

[1] Abu Arab W (2012). Solitary fibrous tumours of the pleura. Eur J Cardiothorac Surg.

[2] Vejvodova S, Spidlen V, Mukensnabl P, Krakorova G, Molacek J, Vodicka J (2017). Solitary fibrous tumor - Less common neoplasms of the pleural cavity. Ann Thorac Cardiovasc Surg.

[3] El-Naggar AK, Ro JY, Ayala AG, Ward R, Ordóñez NG (1989). Localized fibrous tumor of the serosal cavities. Immunohistochemical, electron-microscopic, and flow-cytometric DNA study. Am J Clin Pathol.

[4] Chick JFB, Chauhan NR, Madan R (2013). Solitary fibrous tumors of the thorax: nomenclature, epidemiology, radiologic and pathologic findings, differential diagnoses, and management. AJR Am J Roentgenol.

[5] Rena O, Filosso PL, Papalia E, Molinatti M, Di Marzio P, Maggi G, Oliaro A (2001). Solitary fibrous tumour of the pleura: surgical treatment. Eur J Cardiothorac Surg.

[6] Boyer-Duck E, Dajer-Fadel WL, Hernández-Arenas LÁ, Macías-Morales MP, Rodríguez-Gómez A, Romo-Aguirre C (2018). Pierre-Marie-Bamberger syndrome and solitary fibrous tumor: a rare association. Asian Cardiovasc Thorac Ann.

[7] Piórek A, Kowalski D, Płużański A, Szołkowska M, Wągrodzki M, Koseła-Paterczyk H, Krzakowski M (2019). Solitary fibrous tumour along with non-small-cell lung cancer and Doege-Potter syndrome. Kardiochir Torakochirurgia Pol.

[8] Han G, Zhang Z, Shen X (2017). Doege-Potter syndrome: a review of the literature including a new case report. Medicine.

[9] Kalebi AY, Hale MJ, Wong ML, Hoffman T, Murray J (2009). Surgically cured hypoglycemia secondary to pleural solitary fibrous tumour: case report and update review on the Doege-Potter syndrome. J Cardiothorac Surg.

[10] Szkorupa M, Klein J, Bohanes T, Neoral C, Chudácek J (2010). Solitary fibrous tumor of pleural cavity. Rozhl Chir.

[11] Sun ZG, Wang Z, Zhang M (2011). A 70-year-old man with hypoglycemia, clubbing of fingers and toes, and a large mass of the right hemithorax. Chest.

[12] Lococo F, Cesario A, Cardillo G (2012). Malignant solitary fibrous tumors of the pleura: retrospective review of a multicenter series. J Thorac Oncol.

[13] Dedrick CG, McLoud TC, Shepard JA, Shipley RT (1985). Computed tomography of localized pleural mesothelioma. AJR Am J Roentgenol.

[14] Liu J, Cai C, Wang D (2010). Video-assisted thoracoscopic surgery (VATS) for patients with solitary fibrous tumors of the pleura. J Thorac Oncol.

[15] Tsuro K, Kojima H, Okamoto S (2006). Glucocorticoid therapy ameliorated hypoglycemia in insulin-like growth factor-II-producing solitary fibrous tumor. Intern Med.

[16] Roith DL (1999). Tumor-induced hypoglycemia. N Engl J Med.

[17] Steigen SE, Schaeffer DF, West RB, Nielsen TO (2009). Expression of insulin-like growth factor 2 in mesenchymal neoplasms. Mod Pathol.

[18] Hojo S, Fujita J, Yamadori I (1997). Hepatocyte growth factor and digital clubbing. Intern Med.

[19] Liu CC, Wang HW, Li FY (2008). Solitary fibrous tumors of the pleura: clinicopathological characteristics, immunohistochemical profiles, and surgical outcomes with long-term follow-up. Thorac Cardiovasc Surg.

[20] Santos RS, Haddad R, Lima CE (2005). Patterns of recurrence and long-term survival after curative resection of localized fibrous tumors of the pleura. Clin Lung Cancer.

